# A Mass Spectrometry Strategy for Protein Quantification Based on the Differential Alkylation of Cysteines Using Iodoacetamide and Acrylamide

**DOI:** 10.3390/ijms25094656

**Published:** 2024-04-25

**Authors:** Dávid Virág, Gitta Schlosser, Adina Borbély, Gabriella Gellén, Dávid Papp, Zoltán Kaleta, Borbála Dalmadi-Kiss, István Antal, Krisztina Ludányi

**Affiliations:** 1 Department of Pharmaceutics, Semmelweis University, Hőgyes Endre utca 7., H-1092 Budapest, Hungary; virag.david@semmelweis.hu (D.V.); kiss.borbala@semmelweis.hu (B.D.-K.); antal.istvan@semmelweis.hu (I.A.); 2 MTA-ELTE Lendület Ion Mobility Mass Spectrometry Research Group, Institute of Chemistry, Faculty of Science, ELTE Eötvös Loránd University, Pázmány Péter sétány 1/A, H-1117 Budapest, Hungary; gitta.schlosser@ttk.elte.hu (G.S.); adina.borbely@ttk.elte.hu (A.B.); gabgellen@staff.elte.hu (G.G.); david.papp@ttk.elte.hu (D.P.); 3 Hevesy György PhD School of Chemistry, Institute of Chemistry, ELTE Eötvös Loránd University, Pázmány Péter sétány 1/A, H-1117 Budapest, Hungary; 4 Department of Organic Chemistry, Semmelweis University, Hőgyes Endre utca 7., H-1092 Budapest, Hungary; kaleta.zoltan@semmelweis.hu

**Keywords:** protein quantification, high-resolution mass spectrometry, alkylation, iodoacetamide, acrylamide, human serum albumin

## Abstract

Mass spectrometry has become the most prominent yet evolving technology in quantitative proteomics. Today, a number of label-free and label-based approaches are available for the relative and absolute quantification of proteins and peptides. However, the label-based methods rely solely on the employment of stable isotopes, which are expensive and often limited in availability. Here we propose a label-based quantification strategy, where the mass difference is identified by the differential alkylation of cysteines using iodoacetamide and acrylamide. The alkylation reactions were performed under identical experimental conditions; therefore, the method can be easily integrated into standard proteomic workflows. Using high-resolution mass spectrometry, the feasibility of this approach was assessed with a set of tryptic peptides of human serum albumin. Several critical questions, such as the efficiency of labeling and the effect of the differential alkylation on the peptide retention and fragmentation, were addressed. The concentration of the quality control samples calculated against the calibration curves were within the ±20% acceptance range. It was also demonstrated that heavy labeled peptides exhibit a similar extraction recovery and matrix effect to light ones. Consequently, the approach presented here may be a viable and cost-effective alternative of stable isotope labeling strategies for the quantification of cysteine-containing proteins.

## 1. Introduction

In the past few decades, mass spectrometry (MS) has emerged as the number one tool in protein quantification. Currently, a wide range of proteomic approaches are available for this purpose, which can be classified into two major groups: label-free and label-based methodologies. Label-free quantification (LFQ) measures the correlation between the abundance of a protein and its signal intensity without employing any labeled analogue that would reduce systematic and random biases. The most common LFQ strategies are spectral counting and an extracted ion chromatogram (EIC or XIC), where the former is based on counting the number of times that all peptides corresponding to the same protein are sequenced and the latter relies on determining the chromatographic peak area of precursor ions within a defined *m*/*z* window [1,2,3]. LFQ offers relatively low costs and does not require labor-intensive labeling workflows [4,5]. Moreover, it is on a continuous rise owing to recent developments in high-resolution instruments, new acquisition schemes, and data processing software [6,7]. It is, therefore, the ideal way to reveal relative changes in the abundance of a large set of proteins across multiple samples. On the contrary, the value of label-free approaches is still considered limited when absolute amounts or concentrations need to be determined, e.g., in pharmacokinetic studies and the risk assessment of protein biopharmaceuticals [8,9].

In label-based quantification, a known amount of the labeled (mass-tagged) version of the analyte(s) of interest is introduced into the samples as the internal standard (IS) prior to the analysis. An abundance of the protein is then determined based on the signal intensity ratio of the unlabeled/labeled peptide pairs [10]. Ideally, the IS exhibits the same physicochemical properties as the analyte in order to experience similar recovery losses and matrix effects, while providing sufficient mass differentiation, which is generally achieved by stable isotope labeling. Isotopic labels can be incorporated into the proteins and peptides at different levels of the expression/sample handling process. In the Stable Isotope Labeling by Amino Acids in Cell Culture (SILAC) approach, heavy amino acids are added to the growth medium of a cell culture [11,12,13,14]. This has the advantage of labeling every newly expressed protein that contains the specific amino acids. On the other hand, it can only be used in expression systems, rather than in clinical samples [11]. Similarly, “heavy” amino acids are used in the chemical synthesis of peptides and proteins, which is often referred to as Isotope Dilution Mass Spectrometry (IDMS) [15]. IDMS is widely considered the most reliable quantitative approach; however, it was demonstrated that digestion-based imprecisions may occur due to adding the peptide standard at the end of the process [16]. Although this phenomenon can be partially mitigated using digestible peptides or proteins, their widespread application is limited by their accessibility and high cost. Quantitative strategies relying on the post-harvest isotope labeling have gained considerable popularity as well. Their major advantage over the IDMS is that they do not require custom-synthesized surrogate analytes, because every peptide or protein containing the specific functional group can be derivatized by means of commercially available labeling kits. In the Isobaric Tags for Relative and Absolute Quantitation (iTRAQs) and Tandem Mass Tag (TMT) strategies, lysine amino acids and the *N*-termini of digested peptides from different sources can be tagged with chemical moieties that are identical in mass [17,18,19,20,21]. After combining the samples, the relative abundances of the isobarically labeled peptides are determined by MS/MS experiments, where the reporter ions of different masses are revealed. The compatibility of the iTRAQs with cysteinyl peptide enrichment was also demonstrated to reveal changes in low abundance proteins [22]. Another popular way to introduce stable isotope labeling is Isotope-Coded Affinity Tagging (ICAT), in which cysteines are capped with modified alkylating agents containing a biotin group and a heavy intermediate chain. Thereafter, the proteins are quantitatively combined, digested, and isolated using affinity chromatography [23,24].

To date, all label-based quantitative proteomic strategies rely on the application of stable isotope labels. This work presents a novel approach based on the differential alkylation of cysteine-containing proteins. Instead of using the isotopically labeled counterpart of the derivatizing agent, we applied iodoacetamide (IAM) and acrylamide (AA), upon which reaction with free cysteine amino acids two products are yielded that differ only in a methylene group. After combining them, the heavy labeled cysteinyl peptides generated by enzymatic digestion can serve as internal standards for their light counterparts (or vice versa). Since alkylation using AA is performed under the same conditions as with IAM, this strategy can be completely integrated into standard bottom–up or middle–down proteomic workflows without the need for any extra time-consuming or labor-intensive steps. Furthermore, the labeling step is carried out at the beginning of the sample preparation procedure, lowering the chance of any measurement errors caused by differences in the processing or digestion efficiency [25,26,27,28].

## 2. Results

**Figure 1:**
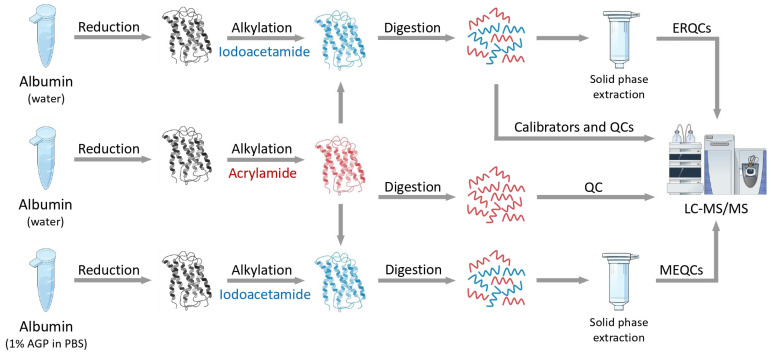
Illustration of the workflow. Unlabeled proteins are marked in grey, while the IAM- and AA-lebeled proteins and peptides are marked in blue and red, respectively.

The goal of this current study was to explore the potential that lies in IAM/AA differential alkylation for the quantitative analysis of cysteine-containing proteins. In this design, AA-derivatized peptides served as internal standards to avoid systematic and random measurement errors. Considering that AA is not a routinely used agent for protein alkylation and the mass differences are not provided by stable isotope-labeled analogues that have nearly identical physicochemical properties to the non-labeled counterparts, several critical questions were addressed including the alkylation efficiency, overalkylation, the effect of differential alkylation on peptide retention times, the extraction recovery, and the matrix effects. Three signature cysteine-containing tryptic peptides of human serum albumin were chosen to monitor the feasibility of the approach: LVRPEVDVMCTAFHDNEETFLK (peptide01), QNCELFEQLGEYK (peptide02), and SHCIAEVENDEMPADLPSLAADFVESK (peptide03). The analytes were selected to cover a relatively broad range in terms of the length of the amino acids to keep track of how the differential alkylation affects the retention times and extraction recoveries of the peptides with various molecular weights. The typical peptide length produced by tryptic digestion is 8–15 residues [29]. While this covers the 13 amino acid-long peptide02, the other two peptides consisting of 21 (peptide01) and 27 (peptide03) amino acid residues are out of this range and overlap with the size of the products generated by the limited proteolysis. The quantitative performance of the strategy was assessed by determining the concentrations of quality control (QC) against the calibration curves based on the peak area ratio of the light and heavy peptide pairs. Two levels of QCs were used, one in the lower (LQC) and one in the upper range of the calibration curve (HQC). Two additional LQC batches were also prepared to monitor the extraction recovery (ERQC) and matrix effect (MEQC), which were subjected to C18 solid phase extraction cleanup. Also, MEQC samples were made in and diluted with 1% α1-acid glycoprotein (AGP) in phosphate buffered saline (PBS) to keep track of potential interferences arising from the complex matrix. The amount of 0.2–4% of albumin in the water or PBS is generally used to mimic complex biofluids, such as the human serum or cerebrospinal fluid, and serve as a surrogate matrix to prepare the calibration and QC samples [25,26,27]. Since albumin is the analyte in this work, AGP, another abundant serum protein, was used to mimic biofluids. To monitor the labeling efficiency, another HQC sample containing only the AA-labeled protein was prepared. The workflow scheme of this study is illustrated in Figure 1.

The popularity of iodoacetamide as a protein-alkylating reagent is largely due to the fact that the reaction goes to completion in a relatively short time under mild conditions (typically room temperature for 20–30 min in darkness), leaving no unalkylated peptides in the sample. Therefore, a fundamental requirement of the differential labeling agent is to have a similar property to avoid systematic measurement errors. The alkylation efficiency was assessed using a HQC sample containing only AA-labeled peptides. The monoisotopic *m*/*z* of the most abundant charge state of each peptide was monitored with an accuracy of 0.1 units throughout the chromatogram (see Figure S1). The peaks that did not reach 0.5% of their labeled counterparts in terms of the area were ignored. Since no peaks reached that value, the acrylamide alkylation can be considered complete indicating that the same reaction conditions can be applied as for iodoacetamide. Peptides are prone to be alkylated primarily at the N-terminus, in addition to cysteines, resulting in overalkylation [30]. This phenomenon was tracked on the LQC and HQC samples in a similar manner as the alkylation efficiency. Since the IAM- and AA-labeled proteins were combined prior to the enzymatic digestion, several products can potentially be generated. Therefore, doubly IAM-labeled, doubly AA-labeled, and peptides containing both labels were monitored. No peaks indicating an extra carbamidoethyl group were detected. On the other hand, doubly carbamidomethylated versions of all the three peptides were found. The peak areas of these by-products were determined and expressed as a percentage of their monoalkylated counterparts to estimate their relative abundance. All the values were below 2.2%.

**Figure 2:**
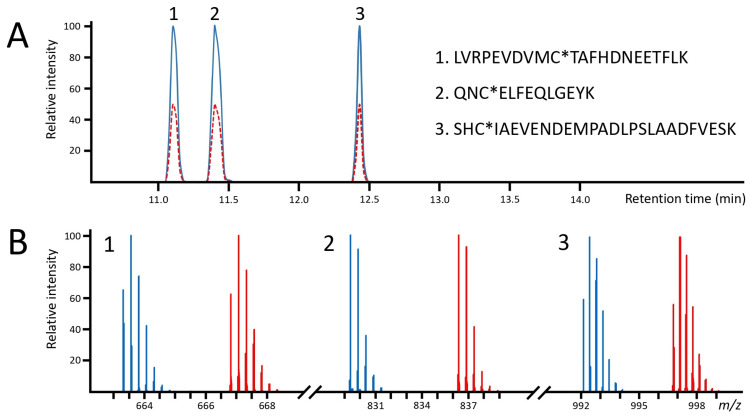
Extracted ion chromatograms (**A**) and mass spectra (**B**) of IAM- and AA-labeled peptides. The blue lines represent the IAM-labeled peptides and the solid and dashed red lines represent the AA-labeled peptides. The ion chromatograms of the heavy peptides were normalized to 50% of the intensity of the light peptides to promote visibility.

In all the quantitative measurements, the peptides were separated with a 22 min gradient chromatography method. Under such conditions, the differentially labeled peptides entirely coeluted (Figure 2A). For the quantitative measurements, an ion chromatogram of the most abundant isotopic peak of the IAM- and AA-labeled peptides was extracted and integrated. As illustrated in Figure 2B, the 14.0157 Da mass difference between the IAM and respective AA label provides a complete separation of the isotopic peak clusters; therefore, no correction equation was needed to calculate the ratios.

**Figure 3:**
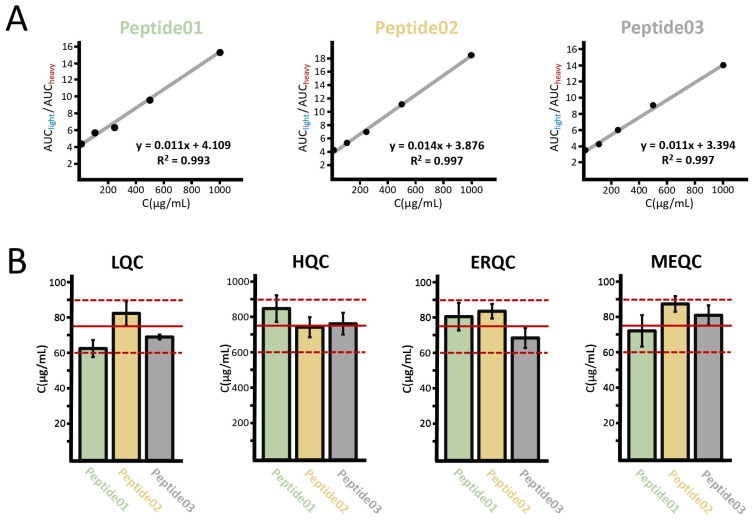
Calibration curves (**A**) and quantification results (**B**) of three peptides. The solid red lines on the bar graphs show the target value, while the dashed red lines represent a 20% tolerance. Peptide01 refers to LVRPEVDVMCTAFHDNEETFLK, peptide02 refers to QNCELFEQLGEYK, and peptide03 refers to SHCIAEVENDEMPADLPSLAADFVESK.

To generate the calibration curves, the area under curve (AUC) ratios of the peptide pairs were plotted as a function of the nominal concentration of the analyte. It is apparent from Figure 3A that the calibration curves showed excellent linearities between 10 and 1000 µg/mL with a determination coefficient (R^2^) of 0.993 or higher. To determine the accuracies (the back-calculated concentration expressed as a percentage of the nominal concentration) and precisions (the relative standard deviation), the concentrations of the triplicate analyses were calculated based on the calibration curves, then averaged. In the LQC and HQC samples, the accuracies were between 83.2 and 112.5%, respectively, while the coefficient of the variation range was 1.9–8.8% (see Figure 3B). In the ERQC and MEQC samples, the accuracies were between 91.1 and 116.0% with precisions ranging between 4.9 and 12.2% indicating that neither the solid phase extraction cleanup nor the potential coeluting peptides considerably affect the quantitative performance of the method (see Figure 3B).

**Figure 4:**
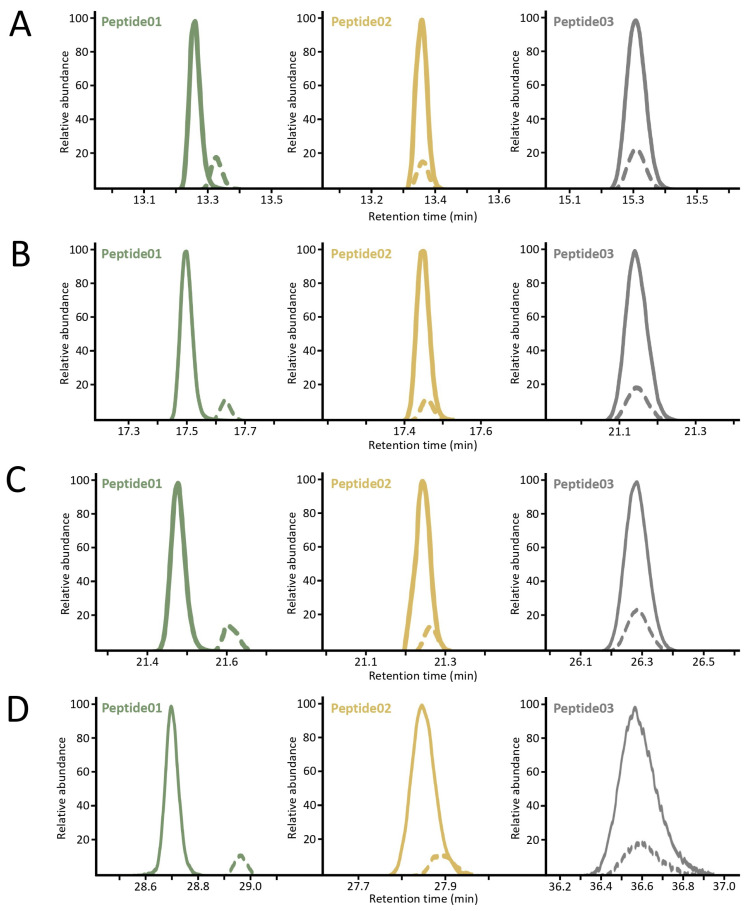
The chromatographic retention behavior of the IAM- and AA-labeled peptides in the 30 min (**A**), 45 min (**B**), 60 min (**C**), and 90 min (**D**) gradient methods. The solid lines represent the IAM-labeled peptides, while the dashed lines represent the AA-labeled peptides. Peptide01 refers to LVRPEVDVMCTAFHDNEETFLK, peptide02 refers to QNCELFEQLGEYK, and peptide03 refers to SHCIAEVENDEMPADLPSLAADFVESK.

Considering that the maximum correction of the matrix effects can be expected if the chromatographic peaks of the analyte and the internal standard completely overlap, the effect of the chromatography on the retention was further tested by means of different gradient methods. Briefly, the organic solvent phase was extended to get a 30, 45, 60, and 90 min gradient method (see Table S1 for detailed settings). Compared to carbamidomethylation, carbamidoethylation resulted in an increase in the retention times in all cases. However, the differential alkylation did not affect the retention of the peptides to the same extent. In the 30 min method, the light and heavy versions of the peptide01 were slightly separated, only a minimal overlap could be observed in the 45 and 60 min method, and they were baseline separated in the 90 min gradient. In the case of the peptide02, a slight shift was observed in all methods. On the other hand, the differently labeled versions of the peptide03 showed complete coelution with all gradient methods. The chromatograms are depicted in Figure 4.

**Figure 5:**
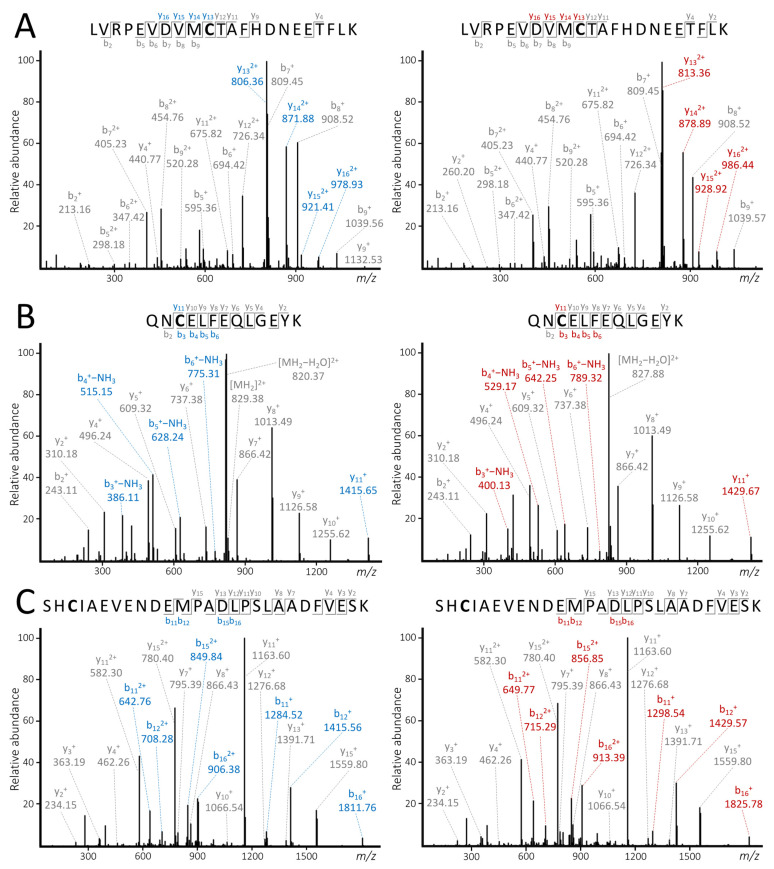
Tandem MS spectra of peptide01 (**A**), peptide02 (**B**), and peptide03 (**C**). The spectra generated from the IAM-labeled peptides are on the left, while the spectra generated from the AA-labeled peptides are on the right. The blue (light) and red (heavy) captions refer to label-specific fragment ions. The non-specific fragments are marked in grey.

In the targeted tandem MS experiments, collision energies (CE) were individually optimized to generate the most abundant product ions containing the labeled cysteine residue. The optimized CEs were 24, 38, and 37 V for peptide01, peptide02, and peptide03, respectively (the detailed settings for the tandem MS experiments are presented in Table S2). The light and heavy versions of each peptide were then subjected to the same CE. As demonstrated in Figure 5, the differential labeling had no apparent effect on the fragmentation pattern of the differentially labeled counterparts. Furthermore, a set of label-specific b and/or y fragment ions were generated depending on the position of the cysteine residue in the peptide.

## 3. Discussion

Among post-harvest labeling strategies, the ones based on the N-terminal derivatization of peptides, such as TMT and iTRAQ, are considered universal. However, targeting cysteine residues seems a viable alternative, since approximately 92–97% of eukaryotic proteins contain cysteine [31]. Furthermore, the alkylation of the sulfhydryl group can be carried out at the intact protein level; therefore, the analyte and the IS can be combined at the beginning of the sample handling, avoiding any source of error due to differential processing. To date, all of these approaches employ isotopically labeled reagents; however, stable isotope labeling is not necessarily a prerequisite to generate reliable ISs for quantitative measurements. Ideally, an IS is expected to track the analyte in all the three distinctive stages of the bioanalytical workflow including the sample preparation, LC separation, and MS measurement [32]. In other words, the IS must represent the analyte in terms of extraction recovery, chromatographic retention behavior, and ionization efficiency, while providing adequate mass differentiation. It is, nonetheless, reported that the methods based on stable isotope labeling do not always meet all the relevant criteria. For example, chromatographic retention times of the heavy and light peptides can be markedly different, and the mass difference is far from the ideal, resulting in overlapping isotope clusters [10].

This present pilot study aimed to answer whether proper ISs can be prepared without the means of isotope labels. The IAM/AA differential alkylating system was used for this purpose. This approach was tested on three tryptic peptides of human serum albumin to validate the results. Applying the typical reaction conditions of protein alkylation to prepare carbamidoethylated peptides, the unlabeled counterpart was practically absent in the control samples, indicating excellent labeling efficiency. This is of crucial importance, because it means the protein carrying the heavy label is equal to the total protein amount in the IS, and there is no need to do any corrections with the labeling efficiency. No overalkylated peptides were detected in the QC samples with the extra AA-label, designating a good reaction selectivity. On the contrary, a relatively low level of IAM overalkylation was experienced, which could be avoided by quenching the excess IAM with DTT prior to the enzymatic digestion to further improve the quantitative performance of this approach [30].

While in a relatively short, 22 min gradient method, the chromatographic peaks of all three peptide pairs completely overlapped, a slight shift in the retention times was observed in the case of the 13-amino acid peptide (peptide02), and baseline separation was achieved for the 22-amino acid peptide (peptide01) when longer gradients were used. On the other hand, the IAM- and AA-labeled 27-amino acid peptide (peptide03) showed no difference in retention. It is known that the key drivers of peptide retention in a reversed-phase chromatography are hydrophobicity and peptide length [33]. It has also been reported that AA-labeled peptides exhibit a higher retention than their IAM-labeled counterparts when formic acid is present in the mobile phase [34]. It might be assumed that the effect of the differential alkylation on retention time shifts is inversely proportional to the peptide length, considering that the single methylene group difference negligibly modifies the retention behavior of large peptides. However, this is not completely justified by the present results, since the light and heavy versions of the 22-amino acid peptide were resolved more than the 13-amino acid ones in the longer chromatographic runs. A plausible explanation for this is that the relative change in polarity generated by the differential alkylation is more pronounced in the hydrophilic peptide01 containing two arginine residues. This may mean that the IAM- and AA-labeled hydrophobic peptides are more likely to coelute. Consequently, the hydrophobic properties of the analytes should be taken into account when selecting candidates for quantification.

The replicate analysis of the QC samples revealed that in most cases this approach met the criteria of the relevant regulatory guidelines, which typically require ≤15% in terms of accuracy and precision [26]. Since the results of the ERQC and MEQC samples were also within the acceptance criteria, it can be concluded that the IAM- and AA-labeled counterparts have a nearly identical extraction recovery and have a similar response to the matrix effects. Although developing MS2-based quantitative experiments was out of the scope of the present study, the results of the targeted MS/MS experiments demonstrated that the differential labeling has no effect on the fragmentation behavior of the peptides. Moreover, several relatively abundant label-specific b and/or y ions were generated during the fragmentation to support establishing completely selective transitions for quantification based on SRM (Selected Reaction Monitoring) and MRM (Multiple Reaction Monitoring).

The approach presented here provides an easy, quick, and noticeably cost-effective way for the label-based quantification of a wide range of proteins. On the other hand, it has a few limitations. First, sample multiplexing is not possible. Second, the correcting effect of the IS is not guaranteed if the light and heavy versions of the peptides do not coelute, which occurred when relatively long gradient programs were used. However, this issue could be most likely solvable in middle–down proteomic applications, in which the effect of the differential alkylation on the retention might be negligible on the large peptides generated by limited proteolysis. Also, alternative reagents may be tested to find the labeling agent pairs that have similar physicochemical properties, while providing an adequate mass difference. Consequently, it seems that a reasonable future direction is to extend this study to alternative proteolytic enzymes (AspN, GluC, and ArgC) and to test a large set of alkylating agents.

## 4. Materials and Methods

### 4.1. Chemicals

Albumin from human serum (≥99%), α1-acid glycoprotein from human plasma (≥99%), trypsin from porcine pancreas (proteomics grade), DL-dithiothreitol (DTT) (≥99.5%), iodoacetamide (≥99%), ammonium bicarbonate (≥99%), LiChrosolv^®^ water, LC-MS grade acetonitrile, phosphate buffered saline (PBS), and formic acid were purchased from Merck (Darmstadt, Germany). RapiGest SF surfactant and Leucine enkephalin were obtained from Waters (Milford, MA, USA). Acrylamide (≥99%) was obtained from Acros Organics (Antwerp, Belgium). Water of Milli-Q purity was prepared with a Simplicity^®^ Water Purification System (Merck Millipore, Burlington, MA, USA).

### 4.2. Sample Preparation

Separate stock solutions of 2 mg/mL were prepared for the calibrators, QC samples, and ISs by reconstituting lyophilized human serum albumin in Milli-Q water. The calibration and QC standards were then serially diluted from their stock solutions with water. The calibrator concentrations were as follows: 1000, 500, 250, 100, and 10 µg/mL. Two levels of QC standards of 750 µg/mL (HQC) and 75 µg/mL (LQC) were also prepared. Two additional batches of the LQC samples were prepared to assess the extraction recovery (ERQC) and matrix effect (MEQC), where the latter was prepared in a surrogate matrix consisting of 1% AGP in PBS. Another HQC sample was prepared, in which the protein was only alkylated with AA. The calibrators were processed and analyzed in duplicates and the QCs in triplicates. The calibration and QC samples were processed in 20 µL aliquots, while 175 µL of IS were prepared in a similar manner. Briefly, a RapiGest SF solution of 0.2% was added to the sample to reach a final concentration of 0.018%, followed by the addition of 100 mM of DTT in the molar ratio to the protein of 250:1, before the concentration was incubated at 60 °C for 30 min. After cooling down the samples, 200 mM of ammonium bicarbonate was added to obtain a final concentration of 21 mM. IAM (100 mM) was added to the samples and AA (100 mM) to the IS, both in the ratio of 500:1 to the protein, then kept at 20 °C for 30 min in the darkness. After the alkylation, the IS was diluted to 275 µL with 200 mM of ammonium bicarbonate, before all the test samples were spiked with 10 µL of the resulting solution, receiving approximately 10 µg of the differentially alkylated protein. The digestion was initiated by adding trypsin at a 1:10 enzyme to substrate ratio and incubated at 37 °C for 12 h. The digestion was quenched by adding formic acid (FA) to a final concentration of 2% (*v*/*v*) and incubated for an additional 30 min. The samples were then dried in a vacuum centrifuge (2000 rpm, 50 °C, 4 h).

The ERQC and MEQC samples were cleaned up using Pierce™ C18 spin columns (Thermo Fisher Scientific, Waltham, MA, USA) based on the manufacturer’s protocol. Apart from with the sample loading, each step was performed in two cycles, where every cycle was followed by centrifugation (1500 rpm, 20 °C, 1 min). First, the resin was activated with 200 µL of 50% acetonitrile (ACN) and equilibrated with 200 µL of 5% ACN (0.5% FA). After changing the microcentrifuge tube, the digest dissolved in 50 µL of 5% ACN (0.5% FA) was added to the resin, and the flowthrough was reapplied one more time. The column was washed with 200 µL of 5% ACN (0.5% FA), and the peptides were eluted with 50 µL of 70% ACN into a new microcentrifuge tube, dried, and kept at −20 °C until the analysis.

### 4.3. LC-MS/MS and Data Analysis

The dried samples (both the raw digested and solid phase extraction purified peptides) were dissolved in 15 µL of 5% ACN containing 0.1% of FA. In total, 4.5 µL of the resulting solution was injected to a Waters Acquity I-Class UPLC system (Waters Corporation, Milford, MA, USA). The peptides were separated on a Waters Acquity Premier CSH column (150 mm × 1 mm, 1.7 μm). The autosampler and the column were maintained at 8 °C and 45 °C, respectively. Mobile phase A was water (0.1% FA), and B was ACN (0.1% FA). The gradient program at a flow rate of 20 µL/min was as follows: 0–0.5 min, 5% B; 0.5–18 min, 5–60% B; 18–18.5 min, 60–85% B; 18.5–21.5 min, 85% B; 21.5–22 min, 85–5% B; and 22–30 min, 5% B. The details of the extended gradient programs can be seen in Table S1. The MS(/MS) experiments were performed with a Waters Select Series IMS QToF Spectrometer (Waters Corporation, Milford, MA, USA) equipped with a low flow electrospray ionization (ESI) source. The source parameters were as follows: polarity, positive; capillary voltage, 2.7 kV; cone voltage, 40 V; source offset, 10 V; source temperature, 120 °C; desolvation temperature, 400 °C; cone gas flow, 20 L/h; desolvation gas flow, 800 L/h; and nebulizer gas pressure, 6 bar. Leucine enkephalin was used as the lock mass for an accurate determination of the mass. The data acquisition was carried out in the *m*/*z* range of 50–2000 in the V-mode. The fragmentation was performed in the transfer cell. In the MS^E^ experiments, the collision energy (CE) was ramped between 19 and 45 V. In the targeted experiments, the CEs were optimized individually for each peptide (see Table S2). The MS^E^ results were searched with the ProteinLynx Global Server 3.0.3 (PLGS) software against the SwissProt database (accessed in July 2023) using the PLGS search engine. The workflow parameters were as follows: digest reagent, trypsin; fixed modification, carbamidomethyl cysteine; variable modification, methionine oxidation; precursor ion tolerance, 20 ppm; fragment ion tolerance, 30 ppm; number of missed cleavages, 2; false discovery rate, 1%; minimum fragment ion matches per peptide, 3; minimum fragment ion matches per protein, 7; minimum peptide match per protein, 1; low energy threshold value, 200 counts; and high energy threshold value, 20 counts. MassLynx 4.1 (Waters Corporation, Milford, MA, USA), OriginPro 2023 (OriginLab, Northampton, MA, USA), Microsoft Excel version 2312 (Microsoft Corporation, Redmond, WA, USA), and mMass were used for the data analysis, processing, and visualization.

## Data Availability

The data used to support the findings of this study are available from the corresponding author upon request.

## Supplementary Materials

The supporting information can be downloaded at: https://www.mdpi.com/article/10.3390/ijms25094656/s1.
