# Supplementary material for: A Mass Spectrometry Strategy for Protein Quantification Based on the Differential Alkylation of Cysteines Using Iodoacetamide and Acrylamide
Source: Int J Mol Sci. 2024 Apr 25;25(9):4656. doi: 10.3390/ijms25094656 (PMC11083099; doi:10.3390/ijms25094656)

Article

A Mass Spectrometry Strategy for Protein Quantification Based on the Differential Alkylation of Cysteines by Iodoacetamide and Acrylamide

Dávid Virág [1], Gitta Schlosser [2], Adina Borbély [2], Gabriella Gellén [2], Dávid Papp [2,3], Zoltán Kaleta [4], Borbála Dalmadi-Kiss [1], István Antal [1], and Krisztina Ludányi [1,*]

[1] Department of Pharmaceutics, Semmelweis University, Hőgyes Endre utca 7., H-1092 Budapest, Hungary; virag.david@semmelweis.hu (D.V.); kiss.borbala@semmelweis.hu (B.D.K); antal.istvan@semmelweis.hu (I.A)
[2] MTA-ELTE Lendület Ion Mobility Mass Spectrometry Research Group, Institute of Chemistry, Faculty of Science, ELTE Eötvös Loránd University, Pázmány Péter sétány 1/A, H-1117 Budapest, Hungary; gitta.schlosser@ttk.elte.hu (G.S.) adina.borbely@ttk.elte.hu (A.B.); david.papp@ttk.elte.hu (D.P.), gabgellen@staff.elte.hu (G.G.)
[3] Hevesy György PhD School of Chemistry, Institute of Chemistry, ELTE Eötvös Loránd University, Pázmány Péter sétány 1/A, H-1117 Budapest, Hungary
[4] Department of Organic Chemistry, Semmelweis University, Hőgyes Endre utca 7., H-1092 Budapest, Hungary; kaleta.zoltan@semmelweis.hu (Z.K.)
* Correspondence: ludanyi.krisztina@semmelweis.hu

Supplementary Materials

Table S1. Settings for the extended gradient method A (30 min), B (45 min), C (60 min), D (90 min).

B%	Time (min)			
	Method A	Method B	Method C	Method D
5 → 60	0.00-18.00	0.00-33.00	0.00-48.00	0.00-78.00
60 → 85	18.01-18.50	33.01-33.50	48.01-48.50	78.01-78.50
85	18.51-23.50	33.51-38.50	48.51-53.50	78.51-83.50
85→ 5	23.51-24.00	38.51-39.00	53.51-54.00	83.51-84.00
5	24.01-30.00	39.01-45.00	54.01-60.00	84.01-90.00

Table S2. Settings for the targeted MS/MS experiments.

Signature Peptide Sequence	Time Segment (min)	LM and HM Resolution	Scan Freq (sec)	CE (V)	Precursor Ion		
					z	*m/z*	
						Light	Heavy
LVRPEVDVM**C**TAF HDNEETFLK	10.8-11.8	4.7, 15.0	0.3	24	+4	663.6	666.1
QN**C**ELFEQLGEYK	10.9-11.9	4.7, 15.0	0.3	38	+2	829.4	836.4
SH**C**IAEVENDEMP ADLPSLAADFVESK	11.9-12.9	4.7, 15.0	0.3	37	+3	992.5	997.2

Figure S1. Evaluating the alkylation efficiency of acrylamide on Peptide01 (A), Peptide02 (B), and Peptide03 (C). Solid red line represents the detector response when monitoring the theoretical *m*/*z* values of the AA-labeled peptides with 0.1 unit accuracy at the most abundant charge state, while dashed green line shows the theoretical *m*/*z* value of the non-labeled counterpart with 0.1 unit accuracy at the same charge state in a HQC sample.

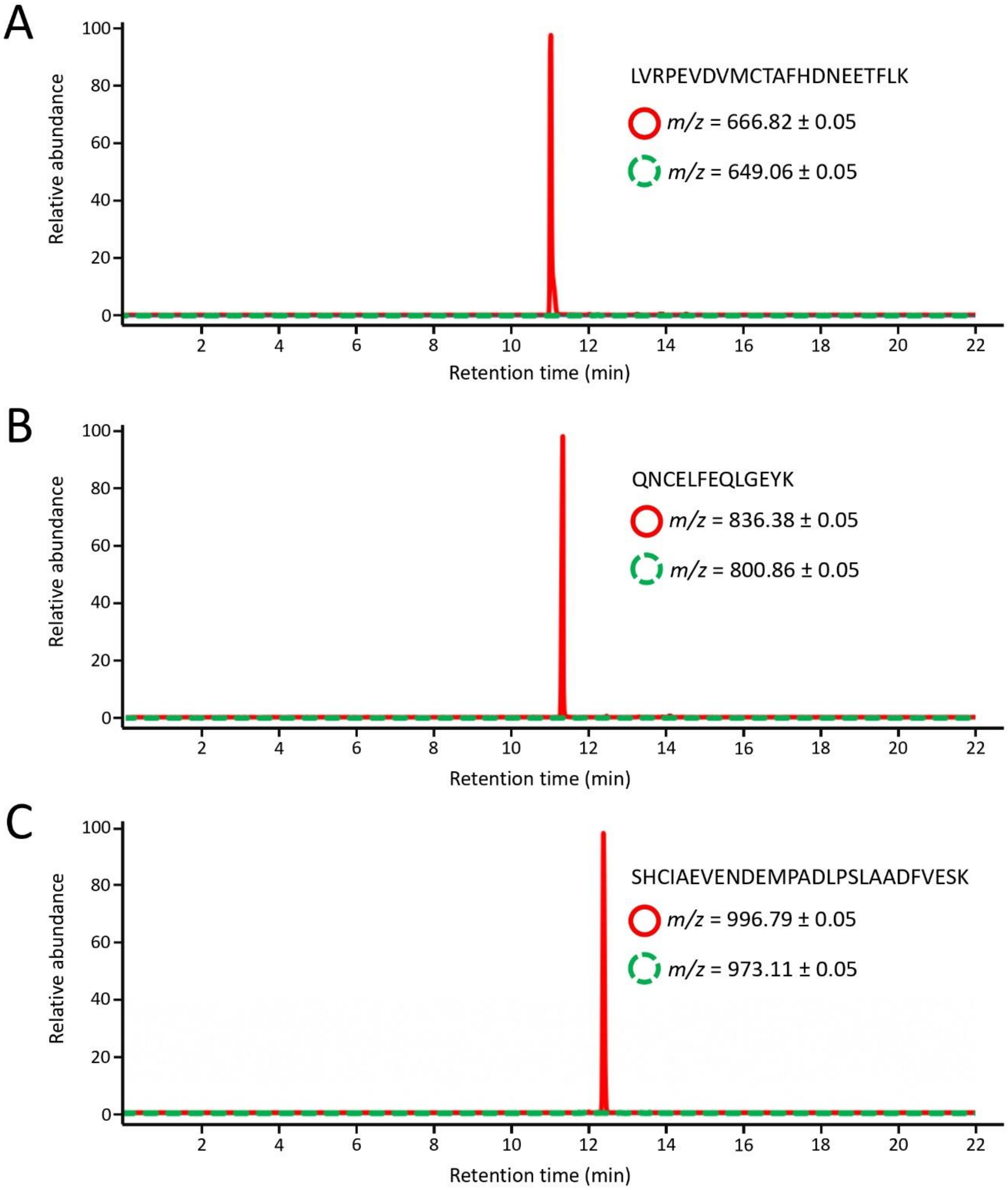

Supplement: Supplementary file 1 [file ijms-25-04656-s001.zip › ijms-2916661-supplementary.pdf]
